# Subcortical Brain Morphometry Differences between Adults with Autism Spectrum Disorder and Schizophrenia

**DOI:** 10.3390/brainsci12040439

**Published:** 2022-03-25

**Authors:** Akila Weerasekera, Adrian Ion-Mărgineanu, Garry Nolan, Maria Mody

**Affiliations:** 1 Department of Radiology, Athinoula A. Martinos Center for Biomedical Imaging, Massachusetts General Hospital, Harvard Medical School, Boston, MA 02115, USA; maria.mody@mgh.harvard.edu; 2 Department of Electrical Engineering (ESAT), STADIUS Center for Dynamical Systems, Signal Processing and Data Analytics, KU Leuven, 3001 Leuven, Belgium; adrian.ionmargineanu@kuleuven.be; 3 Department of Microbiology & Immunology, Stanford University School of Medicine, Stanford, CA 94305, USA; gnolan@stanford.edu

**Keywords:** autism, schizophrenia, MRI, subcortical, hippocampus, amygdala, caudate, social skills, FIQ

## Abstract

Autism spectrum disorder (ASD) and schizophrenia (SZ) are neuropsychiatric disorders that overlap in symptoms associated with social-cognitive impairment. Subcortical structures play a significant role in cognitive and social-emotional behaviors and their abnormalities are associated with neuropsychiatric conditions. This exploratory study utilized ABIDE II/COBRE MRI and corresponding phenotypic datasets to compare subcortical volumes of adults with ASD (*n* = 29), SZ (*n* = 51) and age and gender matched neurotypicals (NT). We examined the association between subcortical volumes and select behavioral measures to determine whether core symptomatology of disorders could be explained by subcortical association patterns. We observed volume differences in ASD (viz., left pallidum, left thalamus, left accumbens, right amygdala) but not in SZ compared to their respective NT controls, reflecting morphometric changes specific to one of the disorder groups. However, left hippocampus and amygdala volumes were implicated in both disorders. A disorder-specific negative correlation (*r* = −0.39, *p* = 0.038) was found between left-amygdala and scores on the Social Responsiveness Scale (SRS) Social-Cognition in ASD, and a positive association (*r* = 0.29, *p* = 0.039) between full scale IQ (FIQ) and right caudate in SZ. Significant correlations between behavior measures and subcortical volumes were observed in NT groups (ASD-NT range; *r* = −0.53 to −0.52, *p* = 0.002 to 0.004, SZ-NT range; *r* = −0.41 to −0.32, *p* = 0.007 to 0.021) that were non-significant in the disorder groups. The overlap of subcortical volumes implicated in ASD and SZ may reflect common neurological mechanisms. Furthermore, the difference in correlation patterns between disorder and NT groups may suggest dysfunctional connectivity with cascading effects unique to each disorder and a potential role for IQ in mediating behavior and brain circuits.

## 1. Introduction

Autism spectrum disorder (ASD) and schizophrenia (SZ) are neuropsychiatric disorders with known phenotypic characteristics, such as social and communication deficits and sensory issues [1,2]. This overlap in the neuropsychological profiles of the disorders, highlights the strong similarities between them, especially when the disorder groups are matched on intelligence quotient (IQ) [3]. Furthermore, deficits of the social brain, a specialized neural network associated with social cognition, appear to be common to both disorders [4,5]. These observations and recent genomic studies seem to suggest that the two disorders may be part of a neurodevelopmental continuum [6,7]. Apart from the similarities, differences exist between the disorders [8,9,10] and delineating the common vs. distinct neural basis of these disorders would be key for understanding their pathophysiology towards developing diagnostic and therapeutical strategies.

Neuroimaging studies of ASD and SZ show common brain abnormalities [11]. In both disorders, irregularities in global brain volumetrics have been previously reported in comparison to neurotypicals (NT). Previous MRI studies report higher total brain volume in early childhood in ASD subjects and lower global gray matter (GM) and white matter (WM) volumes in SZ subjects [12,13]. A meta-analysis study found reduced GM volume in right limbic-striato-thalamic pathway in both conditions [14]. Studies also reported regional brain volume alterations in both conditions: in ASD, reduced volumes were found in the prefrontal cortex (PFC) and temporal regions [15]; similar to ASD, structural alterations in SZ have been found in fronto-temporal regions, anterior cingulate cortex (ACC), amygdala, hippocampus and the insula [16,17]. Functional MRI (fMRI) studies have also shown aberrant activation patterns in fronto-temporo regions and in amygdala in both disorders using mentalizing and basic emotion tasks [18,19]. In addition, both ASD and SZ have been linked with irregularities in white matter connectivity [20,21,22,23]. However, in ASD, most of the studies showing irregular diffusion properties indicating hypo-WM connectivity were conducted in children and, in those focused on adults, the results were inconsistent [11].

A growing area of interest is the alteration of subcortical white matter connectivity in ASD and SZ which is thought to reflect the neurodevelopmental origins of these disorders. Neuroimaging studies have shown changes in the structure and connectivity of white matter tracts involving subcortical regions in the earliest stages of the disorders [22,24,25,26]. While there is evidence that patients with schizophrenia and autism display abnormal subcortical structural and local/global connectivity, the implications for the neurobiology of these disorders remain unclear.

Subcortical structures play a significant role in cognitive and social-emotional behaviors in humans [27,28] (Table 1). Abnormalities of the subcortical structures and their structural/functional connectivity have been associated with neuropsychiatric conditions including ASD and SZ [29,30]. A study conducted using 1571 ASD participants (age range: 2–64 years) and 1651 neurotypical subjects (NT) (age range: 2–56 years) reported smaller subcortical volumes of the pallidum, putamen, amygdala, and nucleus accumbens in the ASD group compared to the NT group [31].

A similar study conducted with 2028 individuals with schizophrenia (age range: 22–43 years) and 2540 NT subjects (age range: 23–42 years) reported lower hippocampal, amygdala, thalamus and accumbens volumes as well as larger pallidum volume in the SZ patients [58]. In general, most studies of ASD and SZ have reported inconsistent volumetric abnormalities of subcortical structures such as the pallidum, accumbens, thalamus, hippocampus, and the amygdala compared to neurotypical subjects [31]. The inconsistencies may be explained by age, IQ-related factors, phenotypic differences within disorders or by various data acquisition and processing methods [59]. In addition, it is also largely unclear how various abnormalities of specific subcortical structures may be associated with cognitive and social-emotional consequences. ASD and SZ show different and, in part, contrasting deficits in social cognition; for example, individuals with ASD and those with SZ typically lack a theory of mind, i.e., the ability to infer the mental states of others. However, in SZ, the ability to attribute mental states can be intensified, i.e., visual/auditory hallucinations. These differences may evidence as distinct subcortical volume alterations when comparing the two disorders.

Therefore, a comparison of subcortical differences between the disorders, compared to NT controls, within a common methodological framework could help delineate overlapping from disorder-specific alterations of brain structure and connectivity. However, to the best of our knowledge, studies to date have focused on the association between subcortical volumetrics and cognitive-social-emotional behavior in individuals with ASD and SZ [19].

Studies that use multi-site datasets from the Autism Brain Imaging Data Exchange (ABIDE) [60], and initiative and schizophrenia data from SchizConnect [61], have the potential to reveal distinct and shared brain abnormalities associated with these disorders. Previously, meta-analyses have been conducted using ABIDE data to investigate the brain volume changes in ASD [59,60,62,63]. However, the conclusions of these studies tend to vary, probably due to factors such as age range and IQ differences of the cohorts, and use of a covariate approach to control for differences between total brain volume (TBV), which assumes the association of TBV and regional volume is linear, whereas it could be allometric or nonlinear [64]. With regard to the SchizConnect database, there appears to be only a few studies using the database to analyze subcortical volumetrics or their association to cognitive measures [27,65]. Additionally, previous studies did not explicitly analyze or compare the correlations between subcortical volumes and cognitive functions pertaining to ASD and SZ for insights into the pathophysiology of these disorders. The current study addresses these issues while building on existing work by examining normalized subcortical gray matter volumes and their association to cognitive scores in age, sex, and IQ-matched subjects with and without ASD (ABIDE) and SZ (SchizConnect).

In this study, we utilized the ABIDE II collection’s Barrow Neurological Institute (BNI) database and SchizConnect’s virtual database, Center of Biomedical Research Excellence (COBRE) to compare subcortical structural volumes (basal ganglia: caudate, putamen, pallidum, nucleus accumbens; limbic structures: hippocampus, amygdala, thalamus) and global gray matter, white matter and total brain volumes of adults with ASD, SZ and age and gender-matched neurotypical subjects. Furthermore, we examined the association between the subcortical volumetrics and neuropsychological measures to determine whether the behavioral symptoms of the disorders could be explained by basal ganglia-limbic-behavior association patterns.

Even though pooling multi-site data offers improved reliability and confidence regarding effect size by averaging out different sources of variability, an important confound of combining multi-site MRI data is the potential for site-specific scanner-related effects to introduce systematic error, consequently making the interpretation of results problematic. Furthermore, partial volume effects and image intensity inhomogeneity are known to introduce bias into automated segmentation of images collected using multiple scanners. Therefore, instead of directly comparing between disorders, we first compared each disorder group with its respective neurotypical control group from the same site, for subcortical volumetric differences (“within disorder/database”) and subsequently compared the differences across the databases (between disorders) to determine distinct and shared morphometric differences between ASD and SZ.

## 2. Materials and Methods

### 2.1. Data Collection

No data collection with human subjects took place at the authors’ institutions. Structural MRI data were drawn from the BNI and COBRE, publicly available image repositories ((http://fcon_1000.projects.nitrc.org/indi/abide/abide_II.html (accessed on 20 December 2020), http://schizconnect.org (accessed on 28 December 2020)). These two databases were chosen since they both contain adult structural MR datasets. All participants provided written informed consent and were scanned according to procedures approved by the local Institutional Review Boards (IRB) at each participating institution.

### 2.2. Participants

Autism: ABIDE II database is an aggregate sample of different studies including imaging and behavioral data for individuals with an ASD diagnosis and typically developing peers. Within ABIDE II, we selected the BNI database for the present study (at the time of data retrieval, BNI consisted of 58 ASD and neurotypical adult males): Twenty-nine males with ASD, 18–65 years old (mean: 37.5 years ± 16) and 29 age- and gender matched neurotypical controls, 18–65 years of age (mean: 39.6 years ± 15). All participants were right-handed males. ASD diagnosis was based on the Autism Diagnostic Observation Schedule-2nd edition (ADOS-2) [66] by an expert clinician. All subjects had IQ in the normal range, one standard deviation below the mean or higher, as measured by the Kaufman Brief Intelligence Test-2 (KBIT-2nd edition) [67]. Exclusion criteria for both groups included MRI scanning contraindications and full-scale IQ scores > 1 standard deviation below the mean on the KBIT-2. Neurotypical subjects were also screened for history of psychiatric or neurological disorders (acquired by a self-report of history and current medication use), immediate family members with ASD, or other major medical conditions that would affect brain functioning [68].

Schizophrenia: Data were downloaded from the Center of Biomedical Research Excellence (COBRE) database via SchizConnect. Fifty-one individuals with schizophrenia (41 males), categorized as “schizophrenia strict” (diagnosed according to The Diagnostic and Statistical Manual of Mental Disorders [DSM] IV) ranging in age from 18–65 years (36.9 ± 14) and 51 healthy controls (38 males), in the same age range (37.6 ± 13) were selected. The participants were mostly right-handed: [SZ], right: 43, left: 7, mixed: 1, (control), right:46, left: 2, mixed: 3. Diagnosis was made using the Structured Clinical Interview used for DSM Disorders (SCID). Neurotypical subjects in the COBRE database were excluded if they had a history of neurological disorder, intellectual disabilities, severe head injuries with more than 5 min loss of consciousness, or substance abuse or dependence within the last 12 months [69]. All subjects scored one standard deviation below the mean or higher, as measured by the Wechsler Abbreviated Scale of Intelligence (WASI) II-[70]. Complete details on subject recruitment may be found at http://cobre.mrn.org/ (accessed on 28 December 2020).

### 2.3. Psychological/Behavior Assessment

A variety of neuropsychological measures were used to assess the subjects in the two databases. We focused on social cognition, given its role in social deficits characteristic of both disorders towards our goal of converging on the neurobiology of social cognition and interrogating it for disorder-specific pathways. For ASD, the Social Responsiveness Scale (SRS-2) [71] was used to assess the severity of the ASD symptoms. Social Cognition subscale scores were selected for analysis. For the participants with SZ, we focused on scores on the Mayer-Salovey-Caruso Emotional Intelligence Test (MSCEIT) [72], a measure of social-cognitive ability. In addition, we included full scale IQ measure to exclude concerns of intellectual disability and to control for group differences in assessing brain-behavior associations.

### 2.4. Imaging Data

Downloaded datasets from ABIDE and COBRE included a high resolution T1-weighted structural MRI scan for each of the subjects. MR scanner and structural acquisition parameters varied across sites.

The ABIDE-BNI scans were acquired on a 3T Philips Achieva MRI (Philips Medical Systems, Best, The Netherlands) with a 15-channel receive coil. T1 acquisition sequence: MPRAGE, TR/TE/TI/flip angle = shortest/shortest/900 ms/9°, number of excitations (NEX) = 1 number of slices = 170, Slice voxel size = 1 × 1 × 1 mm^3^, field of view (FOV) = 270 × 252 mm.

The COBRE scans were acquired on a 3T Siemens MAGNETOM TrioTim syngo (Siemens, Erlangen, Germany) with a 12-channel receive coil. T1-weighted images were acquired with a 5-echo multi-echo MPRAGE sequence [TE = 1.64, 3.5, 5.36, 7.22, 9.08 ms, TR/TI = 2530 ms/1200 ms/7°, NEX = 1, slice thickness = 1 mm, voxel size = 1 × 1 × 1 mm^3^, FOV = 256 × 256 mm.

### 2.5. Image Preprocessing

#### Structural Data

All T1-images were manually inspected for quality and motion artifacts. Processing was done using the FreeSurfer v. 7.1.1 ((http://surfer.nmr.mgh.harvard.edu [Boston, USA]) recon-all pipeline with the default settings. In addition to FreeSurfer image segmentation procedures, we assessed and compared the Freesurfer segmentation using the quality outputs from FreeSurfer QAtools ((https://github.com/Deep-MI/qatools-python (accessed on 1 February 2021)) across male neurotypicals from both BNI and COBRE. The quality measures included for SNR, anatomical signal-to-noise ratio in white matter, mWM: mean white matter intensity, voxWM: total number of white matter voxels, standard deviation of white matter intensity (stdWM); and for CNR, contrast-to-noise ratio. In addition, we also compared the estimated total intracranial volume (eTIV) and the non-(eTIV) normalized global volumetrics such as total gray matter (tGM), subcortical gray matter (sGM), cerebral white matter (cWM), cerebrospinal fluid (CSF) of the neurotypicals from the two databases in order to assess potential differences that may relate to site-specific effect. Segmented labels were visually inspected to identify potential segmentation artefacts and manual corrections were applied when needed. Segmentations were discarded from the study if manual corrections were not possible. The FreeSurfer output of subcortical volumes (caudate, putamen, globus pallidus, nucleus accumbens, amygdala, hippocampus, thalamus), and global volumes such as the total gray and cerebral white matter, were used as feature groups in the analysis. These volumes were normalized to eTIV to control for variability due to sex-related as well as individual differences in brain size.

### 2.6. Statistical Analysis

To avoid potential confounds in data analysis due to site-specific scanner-related differences, each disorder group was compared to its respective control group from the same site. An analysis of variance (ANOVA) yielded differences between the groups in global brain and subcortical volume measures. Age was not included as a covariate in this study since age did not differ significantly between groups (Table 2). Follow up comparisons between the groups were performed using the Šidák post hoc test. To identify the relationship between subcortical volumetrics and behavior measures in ASD and SZ, volumes were associated with behavior scores using Pearson’s correlation. Statistical significance was defined as *p* < 0.05. Data were analyzed using GraphPad Prism (9.0.0).

## 3. Results

In this study, all analyses were performed between groups within a database (i.e., ASD vs. ASD-NT (ABIDE II-BNI) and SZ vs. SZ-NT (COBRE) for all brain volumes. No direct comparisons were made between ASD and SZ to avoid potential confounds associated with data acquisition site-related factors.

### 3.1. Quality Assessment between Neurotypical Males in ASD and SZ Databases

We found significant differences in white matter quality metrics viz., SNR, mWM intensity and stdWM between the two neurotypical datasets (Table 3). As for segmentation of global volumes (non-normalized), we found significant differences in tGM and eTIV between groups (Table 3). As such, these site-specific differences between the two neurotypical groups precluded any direct comparisons between the ASD and SZ subjects who also came from the different sites.

### 3.2. Global Brain Volumes between Groups (Disorder vs. Neurotypical)

**Figure 1:**
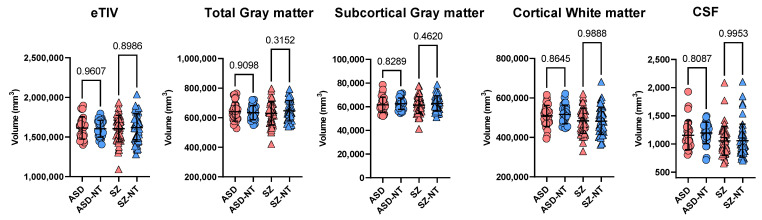
Mean ± standard deviation for non-normalized volumetric measures (in mm^3^) of total gray matter, subcortical gray matter, cortical white matter and CSF for the disorder groups and their respective neurotypical groups (ASD [*n* = 29] and ASD-NT [*n* = 29]; SZ [*n* = 51] and SZ-NT [*n* = 51]). Statistical significance of group differences is indicated numerically. Red circle: ASD, blue circle: ASD-NT, red triangle: SZ, blue triangle: SZ-NT.

Estimated total intracranial volume (eTIV) did not differ significantly between the groups within the two databases. No significant differences in tGM, sGM, cWM or CSF were found between disorder and respective control groups (Figure 1).

**Figure 2:**
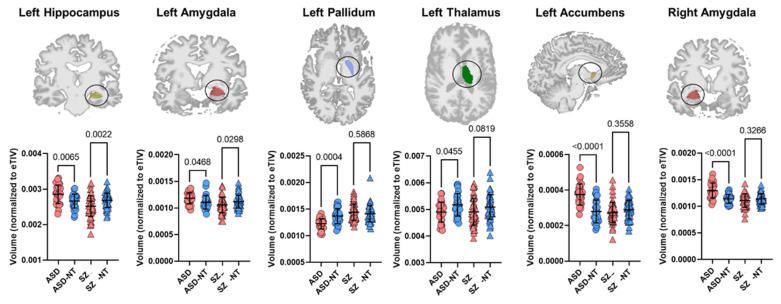
Mean ± standard deviation for eTIV-normalized volumetric measures of subcortical structures in disorder groups and their respective neurotypical groups (ASD [*n* = 29] and ASD-NT [*n* = 29]; SZ [*n* = 51] and SZ-NT [*n* = 51]). Statistical significance is indicated numerically. Red circle: ASD, blue circle: ASD-NT, red triangle: SZ, blue triangle: SZ-NT.

As for disorder-specific findings, the volumes of left pallidum (*p* = 0.0004, Cohen’s d = 1.2) and left thalamus (*p* = 0.0455, Cohen’s d = 0.69) were significantly smaller in ASD and left accumbens (*p* < 0.0001, Cohen’s d = 1.5) and right amygdala (*p* < 0.0001, Cohen’s d = 1.32) volumes were significantly larger compared to the neurotypicals. No significant differences were found for these or other subcortical structures specific to SZ compared to its control group (Figure 2).

### 3.3. Correlations between Subcortical Volumes, IQ and Social Cognition

**Figure 3:**
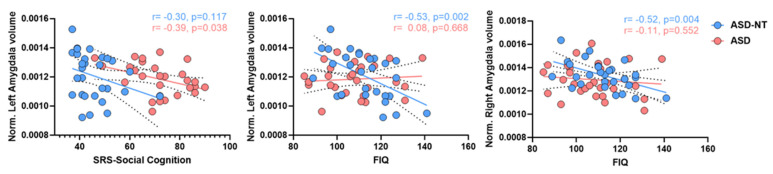
Correlation plots in ASD. (**Left**): correlation between normalized left amygdala volume and SRS-social cognition score in ASD (*r* = −0.39, *p* = 0.038) and ASD-NT (*r*= −0.30, *p* = 0.117). (**Middle**): correlation between normalized left amygdala volume and FIQ score in ASD (*r* = 0.08, *p* = 0.668) and ASD-NT (*r*= −0.53, *p* = 0.002). (**Right**): correlation between normalized right amygdala volume and FIQ score in ASD (*r* = −0.11, *p* = 0.552) and ASD-NT (*r* = −0.52, *p* = 0.004). Solid-lines: regression-line (red = ASD, blue = ASD-NT); dashed-lines indicate the 95% confidence intervals for respective regression lines.

In ASD, a negative correlation was found between left amygdala volume and SRS-social cognition (*r* = −0.39; *p* = 0.038) which was not significant for the ASD-NT group (*r* = −0.30, *p* = 0.1174) (Figure 3). Conversely, we found significant negative correlations between the bilateral amygdala and FIQ in the NT group (left: *r* = −0.53, *p* = 0.0020, right: *r* = −0.52, *p* = 0.004 (Table 4). No other correlations were significant in ASD or SZ datasets.

**Figure 4:**
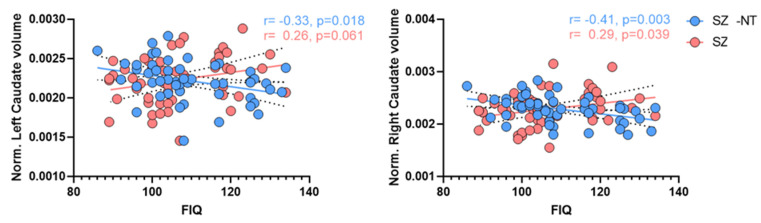
Schizophrenia correlation plots. (**Left**): correlation between normalized left caudate volume and FIQ in SZ (*r* = 0.26, *p* = 0.061) and SZ-NT (*r* = −0.33, *p* = 0.018). (**Right**): correlation between normalized right caudate volume and FIQ score in SZ (*r* = 0.29, *p* = 0.039) and SZ-NT (*r* = −0.41, *p* = 0.003). Solid-lines: regression-line (red = SZ, blue = SZ-NT); dashed-lines indicate the 95% confidence intervals for respective regression lines.

We observed significant correlations between a number of subcortical structures and FIQ in SZ neurotypical group compared to SZ group (Table 5); most of these were negative. In contrast, only two correlations in the SZ group were significant, and both were positive. Bilateral caudate volumes showed significant negative correlations with FIQ (left: *r* = −0.33, *p* = 0.018; right: *r* = −0.41, *p* = 0.003) in SZ-NT group; in contrast, the right and left caudate showed positive (*r* = 0.29, *p* = 0.039) or a trend towards positive (*r* = 0.26, *p* = 0.061) correlations with FIQ in the SZ group (Figure 4). Scores on the MSCEIT showed no correlations in either SZ or NT.

## 4. Discussion

Multi-site studies are increasingly common in neuroimaging research due to the potential for large sample sizes and more robust results. Multi-site MRI datasets provide researchers the ability to compare neuroanatomy across several neurological disorders and test different hypotheses, the pooled data also providing improved statistical power. However, multi-site neuroimaging studies have the potential to introduce noise in the data due to site-specific differences related to the type of scanners and MRI sequences used, as was evident in the quality metric and volume segmentation differences between the NT groups from the two sites in the present study. Consequently, any group differences from a direct comparison between ASD and SZ in the current study could be attributed to site-specific effects. Therefore, between groups direct comparisons were confined to disorder vs. control groups from the same site, while allowing us to indirectly compare the subcortical volumetric differences between the two disorders.

### 4.1. Direct Comparisons

#### 4.1.1. ASD and ASD-NT

In our study, compared to NT, the ASD group showed significant volumetric differences in several subcortical structures in the left-hemisphere including the pallidum, hippocampus, accumbens, thalamus and bilateral amygdala. In the left hemisphere, pallidum and thalamus were characterized by significantly lower volumes, whereas the hippocampus, and accumbens and bilateral amygdala showed higher volumes compared to the NT subjects. Interestingly, the results appear to implicate subcortical structures in the left hemisphere, suggestive of a lateralized dysfunction in ASD, which has been reported previously [73,74,75,76]. Furthermore, we observed a negative correlation between the left amygdala and SRS-social cognition only in the ASD group, and negative correlations between bilateral amygdala and FIQ in the NT group but not in the ASD group (Table 4). It should be noted that bilateral amygdala volumes were significantly increased in the ASD group compared to NT group. The two groups, though, matched on FIQ differed on SRS scores. As such, the difference in volume between the groups may give rise to the different pattern of structure-function associations observed and have implications for white matter. It has been shown that an increase in the number of cortical neurons takes up space needed for axonal connections, thereby resulting in a net decrease in white matter connectivity [77]. Therefore, we can assume that this is true for subcortical structures as well, where larger structures may decrease the cortical/subcortical white matter connectedness leading to atypical cognitive functioning.

Subcortical structural differences have been previously reported in ASD compared to neurotypical controls, though with substantial heterogeneity regarding their direction and magnitude [78,79]. A recent large-scale meta-analysis based on 51 existing datasets reported individuals with ASD to have reduced volumes of the pallidum, putamen, amygdala, and nucleus accumbens compared to controls [59]. However, amygdala volume alterations in ASD have been mixed, with studies reporting increased [80,81,82,83,84,85,86], decreased [87,88,89] or no difference in volume [90] compared to neurotypical controls. Similarly, hippocampal findings show increased and decreased volumes in ASD regardless of age [83,87,91,92,93]. Based on evidence from neuropathology and neuroimaging studies in humans and animals, the basal ganglia are believed to play a role in the neurobiology of autism. Overall enlargement of the basal ganglia in ASD has been reported compared to neurotypicals [94]; however, the findings have been inconsistent [79,95]. Similarly, conflicting findings exist for the thalamus [96,97,98,99].

The subcortical volumetric findings in our study support the involvement of the basal ganglia, limbic system, and thalamus in ASD. In addition, scores on the SRS social cognition and FIQ appeared to differentially associate with the amygdala in ASD compared to NT controls, suggesting a possible influence of IQ on social skills mediated by the amygdala in ASD. In a previous study conducted with children ages 3–4, bilateral enlargement of amygdala and larger right amygdala volume was associated with slower acquisition of social and communicative skills; furthermore, the same study reported a larger left amygdala volume at ages 3–4 years and predicted improved language outcome at age 6 years [86]. It is possible that early abnormal development of the amygdalae may result in dysregulated connectivity (cortical and subcortical), and asymmetric functional contributions to social skills. Our analysis also yielded a negative association between bilateral amygdala and FIQ in NT but not ASD, hinting at a possible role for intelligence in moderating structure-function relationships as in ASD cognition [100,101].

#### 4.1.2. SZ and SZ-NT

Studies of schizophrenia report significant subcortical morphological abnormalities in patients with the disorder, though there is considerable heterogeneity in the pattern of structural differences across studies [30,102,103,104,105]. In our study, individuals with schizophrenia showed significant volumetric differences in left hemisphere, hippocampus and amygdala, compared to neurotypicals. Both these structures showed decreased volumes compared to the NT group. Similar to our findings, a recent case-controlled meta-analysis with 2028 patients with schizophrenia and 2540 healthy controls showed smaller hippocampus and amygdala in patients compared to healthy controls. The same study also showed smaller accumbens, thalamus as well as larger pallidum, which we did not observe [58].

The striatum, made up of the caudate, putamen and nucleus accumbens, is one of the most essential subcortical components of the cortico-striato-thalamo-cortical circuits. It is involved in integrating and modulating sensory information, important in learning and cognition [106]. In fact, the caudate and putamen receive axonal fibers from the cortex and the intralaminar nuclei of the thalamus, in keeping with their involvement in sensory and cognitive information processing. A previous study in schizophrenia patients reported correlations between reduced putamen volume and deficits in verbal learning, working memory, and higher cognitive function, which are essential for language processing [107]. In our study, we found a positive association of FIQ with right caudate volume, and a positive trend with the left caudate in the SZ group, unlike in the NT group where these associations were found to be negative. The right putamen and bilateral accumbens, too, were correlated, a negative association, but only in the NT group. Taken together, this difference in the association of the caudate, putamen and accumbens with FIQ between the groups suggests a possible role for the striatum, particularly the caudate, in mediating cognitive outcomes in SZ. Recent studies have reported IQ, reasoning and problem-solving skills as positively correlating with the hippocampus, amygdala and nucleus accumbens volumes in patients with schizophrenia [17,93]. Although we found significant group differences in some of these structures, no significant associations were observed between these structures and FIQ in the SZ group; rather, FIQ showed negative associations or a negative trend with left hippocampus and bilateral accumbens in the NT group. Given the role of the hippocampus in learning and memory, the finding raises questions about potentially impaired striatal-hippocampus connections affecting temporal cognition in schizophrenia evident in thought disorder and contextually inappropriate behavior [108,109,110].

### 4.2. Indirect Comparisons: ASD vs. SZ in Relation to NT

In this study, we observed subcortical structural differences in both ASD and SZ; some of these structures implicated in both disorders. Specifically, the left hippocampus and left amygdala volumes were higher in ASD and lower in SZ compared to respective NT.

Subcortical structures, which include the basal ganglia, limbic system and the thalamus, have been shown to be involved in learning and memory, as well as other key functions such as motor control, attention and emotion [35,111,112]. Subcortical structures also have important roles in higher-order executive functions including inhibitory control and working memory through their structural and functional connectivity with prefrontal and temporal regions [113]. ASD and SZ are both neurodevelopmental disorders that share impairments in social behavior and cognitive function [4,5]. However, both are considered distinct disorders, with the symptoms of autism initially showing in early childhood while positive symptoms of schizophrenia typically appear in early adulthood [114]. Our focus on subcortical structures, and their potential association with social-emotional cognition implicated in both disorders, has the potential to offer valuable clinical insights and help guard against diagnostic conflation that has been historically problematic as many young individuals with ASD were thought to have a childhood version of schizophrenia. In this study we found subcortical volumetric associations with scores on the SRS in ASD, but not with the MSCEIT, the only measure available in the social emotional domain in the COBRE database. Despite the vagaries associated with behavioral testing and test measures, our findings of the hippocampus and amygdala volume differences common to both disorders have been previously implicated as having neurobiological basis for social cognition and emotion processing in ASD and SZ patients [18,115]. This may suggest impairment in a specific subcortical-cortical network of social cognition in both disorders.

Overall, our results comparing ASD and SZ with neurotypicals show support for the role of the basal ganglia in these disorders. Considering the spatial proximity and the structural connectivity between the amygdala and hippocampus, opposing volumetric anomalies and their association with cognitive function, as observed in the current study, may well explain the structural and functional connectivity differences in other brain areas in keeping characteristic symptoms of the two disorders.

Finally, our observations of the subcortical volumetric differences and their correlation with behavioral measures in ASD and SZ may suggest potential alterations in the cortical-basal ganglia network. As such, similar volumetric or networkwide alterations may also exist within the limbic system, as we see in our study, a potential consequence of adaptive-compensatory mechanisms over the course of development.

## 5. Conclusions

We acknowledge the limitations of our study. Due to site-specific scanner-related differences in the collection of the ASD and SZ data, a direct comparison between these disorders was not possible. The results should be considered preliminary, given the difference in the sample size of the ASD and SZ datasets. Nevertheless, despite the exploratory nature of the study, the difference in subcortical volumetrics and the pattern of correlation with cognitive behavior in ASD and SZ suggest dysfunctional connectivity with cascading effects unique to each disorder.

In summary, while focusing on complex behaviors in the social domain, paying attention to elemental subtle differences, both structural and functional, that are common across ASD and SZ, may help identify the basis for core characteristics in these disorders. In addition, this approach could guide the development of diagnostic and intervention strategies for autism and schizophrenia based on underlying neurobiological differences.

## Figures and Tables

**Table 1:** Subcortical structures and functions.

Subcortical Structure	Function
Caudate nucleus	Directed movements [32], working memory [33,34], language [35,36], learning [37], Goal-directed action [38,39].
Putamen	Motor skills [40,41], learning [42,43,44]
Pallidum	Voluntary movement [45], reward and motivation [46,47]
Nucleus accumbens	Motivation, reward, locomotor activity, learning, memory [48,49]
Amygdala	Emotional learning [50], memory modulation [51]
Hippocampus	Episodic memory [52,53], response inhibition, spatial cognition [54,55]
Thalamus	Relay sensory signals, arousal and pain regulation, motor, language function, mood and motivation, cognition [56,57]

**Table 2:** Demographics and Clinical Characteristics of ASD, SZ and Neurotypicals.

Parameter	ASD N = 29 Mean (SD)	ASD- NT N = 29 Mean (SD)	*p*-Value	SZ N = 51 Mean (SD)	SZ-NT N = 51 Mean (SD)	*p*-Value
Age (years)	37.5 (16)	39.6 (15)	0.6037	36.9 (14)	37.6 (13)	0.8997
Gender (m/f)	29/0	29/0		41/10	38/13	
FIQ ^a^	107.6 (13)	112.5 (12)	0.1756	106.6 (14)	109.8 (12)	<0.1667
SRS Social Cognition ^b^	73.2 (10)	50.1 (13)	<0.0001	-	-	
MSCEIT ^c^	-	-		44.7 (11)	53.1 (9)	<0.0001

Significance threshold was defined as *p* < 0.05. ^a^ FIQ was measured with KBIT-2nd edition for ASD and with WASI-II for SZ (normal range: 80–120). ^b^ SRS Social Cognition: subscale of the Social Responsiveness Scale. SRS score: 60–90 (mild to severe); 35–60 (normal). ^c^ MSCEIT: Mayer-Salovey-Caruso Emotional Intelligence Test. MSCEIT: NT: 50–100 (developing to competent); <50 (difficulties with emotional cognition).

**Table 3:** FreeSurfer image segmentation quality parameters of ASD-NT and SZ-NT.

	ASD-NT Males N = 29 Mean (SD)	SZ-NT Males N = 38 Mean (SD)	*p*-Value
SNR	21.6 (3)	18.7 (2)	<0.0001
CNR	1.4 (0.08)	1.4 (0.09)	0.9759
voxWM	57,432 (13,963)	53,285 (12,866)	0.2152
mWM	104.3 (0.77)	102.2 (0.95)	<0.0001
stdWM	4.9 (0.60)	5.6 (0.93)	0.0009
tGM	634,018 (47,821)	668,310 (62,118)	0.0159
sGM	62,567 (4839)	64,541 (6135)	0.1567
cWM	516,466 (47,997)	503,904 (61,055)	0.3626
CSF	1195 (190.2)	1111 (308.1)	0.2002
eTIV	1,604,009 (106,800)	1,683,745 (137,571)	0.0117

**Table 4:** Correlations between subcortical volumes and behavior in ASD and ASD-NT.

Correlation	ASD	ASD-TD
Left Amygdala vs. FIQ	*r* = 0.08, *p* = 0.668	*r* = −0.53, *p* = 0.002
Right Amygdala vs. FIQ	*r* = −0.11, *p* = 0.552	*r* = −0.52, *p* = 0.004
Left Amygdala vs. SRS Social Cognition	*r* = −0.39, *p* = 0.038	*r* = −0.30, *p* = 0.117

Red text indicates statistical significance (*p* < 0.05).

**Table 5:** Correlations between subcortical volumes and behavior in SZ and SZ-NT.

Correlation	SZ	SZ-TD
Left Caudate vs. FIQ	*r* = 0.26, *p* = 0.061	*r* = −0.33, *p* = 0.018
Right Caudate vs. FIQ	*r* = 0.29, *p* = 0.039	*r* = −0.41, *p* = 0.003
Right Putamen vs. FIQ	*r* = 0.02, *p* = 0.883	*r* = −0.35, *p* = 0.013
Right Pallidum vs. FIQ	*r* = −0.04, *p* = 0.759	*r* = −0.37, *p* = 0.007
Left Hippocampus vs. FIQ	*r* = 0.15, *p* = 0.289	*r* = −0.32, *p* = 0.021
Left Accumbens vs. FIQ	*r* = 0.23, *p* = 0.110	*r* = −0.27, *p* = 0.057
Right Accumbens vs. FIQ	*r* = 0.21, *p* = 0.213	*r* = −0.27, *p* = 0.053

Red text indicates statistical significance (*p* < 0.05). Blue text indicates trends towards statistical significance.

## Data Availability

Not applicable.
